# STING Millennium Suite: integrated software for extensive analyses of 3d structures of proteins and their complexes

**DOI:** 10.1186/1471-2105-5-107

**Published:** 2004-08-09

**Authors:** Roberto H Higa, Roberto C Togawa, Arnaldo J Montagner, Juliana CF Palandrani, Igor KS Okimoto, Paula R Kuser, Michel EB Yamagishi, Adauto L Mancini, Goran Neshich

**Affiliations:** 1 Núcleo de Bioinformática, Centro Nacional de Pesquisa Agropecuária, Empresa Brasileira de Pesquisa Agropecuária, Campinas, SP, Brazil; 2 Laboratório de Bioinformática, Embrapa/Recursos Genéticos e Biotecnologia, Empresa Brasileira de Pesquisa Agropecuária, Brasília, DF, Brazil

## Abstract

**Background:**

The integration of many aspects of protein/DNA structure analysis is an important requirement for software products in general area of structural bioinformatics. In fact, there are too few software packages on the internet which can be described as successful in this respect. We might say that what is still missing is publicly available, web based software for interactive analysis of the sequence/structure/function of proteins and their complexes with DNA and ligands. Some of existing software packages do have certain level of integration and do offer analysis of several structure related parameters, however not to the extent generally demanded by a user.

**Results:**

We are reporting here about new Sting Millennium Suite (SMS) version which is fully accessible (including for local files at client end), web based software for molecular structure and sequence/structure/function analysis. The new SMS client version is now operational also on Linux boxes and it works with non-public pdb formatted files (structures not deposited at the RCSB/PDB), eliminating earlier requirement for the registration if SMS components were to be used with user's local files. At the same time the new SMS offers some important additions and improvements such as link to ProTherm as well as significant re-engineering of SMS component ConSSeq. Also, we have added 3 new SMS mirror sites to existing network of global SMS servers: Argentina, Japan and Spain.

**Conclusion:**

SMS is already established software package and many key data base and software servers worldwide, do offer either a link to, or host the SMS. SMS (Sting Millennium Suite) is web-based publicly available software developed to aid researches in their quest for translating information about the structures of macromolecules into knowledge. SMS allows to a user to interactively analyze molecular structures, cross-referencing visualized information with a correlated one, available across the internet. SMS is already used as a didactic tool by some universities. SMS analysis is now possible on Linux OS boxes and with no requirement for registration when using local files.

## Background

A need to integrate, visualize and mine large amount of protein structure data is accelerating. In order to accommodate visualization of data originating from several sources and make analysis of protein structure and structural parameters easier, we developed Sting Millennium Suite (SMS). SMS is a web-based suite of programs and databases providing visualization and a complex analysis of molecular sequence and structure for the data deposited at the Protein Data Bank (PDB) [1].

Using SMS it is possible to analyze: sequence to structure relationships, quality of the structure, nature and volume of atomic contacts of intra and inter chain type, relative conservation of amino acids at the specific sequence position based on multiple sequence alignment, indications of Folding Essential Residue (FER) based on relationship of the residue conservation to the intra-chain contacts, Cα – Cα and Cβ – Cβ distance geometry etc.. Specific emphasis in SMS is given to Interface Forming Residues (IFR) – amino acids that define interactive portion of the protein surfaces. SMS may simultaneously display and analyze previously superimposed structures.

Parsing of data from relevant Data Bases (PDB [1], HSSP [2,3], Prosite [4]) is one of the key features of integrated SMS environment for structure/function analysis. SMS also has its own built in data bases: Contacts, Interface Contacts, Surface Accessibility, Dihedral Angles and Secondary Structure Elements [5].

This article is intended to show how Sting Millennium Suite of programs can be useful in the study of protein structure and analysis of its function, emphasizing recent improvement introduced to SMS. The program has extensive built-in instructions and detailed easy-to-use help which user is invited to consult before and during SMS use.

## Results and Discussion

### SMS overview

In addition to basic macromolecular visualization, SMS is capable of identifying and visualizing the macromolecular interfaces as well as showing and analyzing previously aligned structures. SMS also does visualization of amino acids conservation based on multiple sequence alignments, in the context of three-dimensional protein structure, identification of the nature and volume of atomic contacts of intra and inter-chain type, presentation of data about the quality of a given structure etc.. SMS provides number of modules (SMS components (some of which are to be described in details separately)) to conveniently visualize large amount of physical-chemical, structural and biological information about the proteins with known structure. Variety of one-click-away renderings and color schemes helps to visualize bonding interactions and locations of residues of interest, as well as to localize patterns of evolution/conservation. The interactions which occur in the protein or between protein and its inhibitor/substrate, can be analyzed in great details with SMS.

#### Graphical contacts

SMS offers to the user a graphical presentation of inter-atomic contacts established between amino acids in form of the fan. The base point of the fan is the selected amino acid. From the base point a user can detect number of colored lines connecting to other residues (presented by single letter code). Colors of the fan lines follow SMS code of contacts. A specific HTML table displays residue name and number, its pair in contact establishing, type of the contact, distance between contacting atoms and accessibility and entropy of two contacting residues. Such contacts are divided in number of classes: hydrogen bonds, hydrogen bonds with intermediary water molecules, hydrophobic contacts, aromatic ring stacking contacts, electrostatic (attractive and repulsive) contacts and finally disulphide bridges. A special table is built for those interactions across the interface (*IFR Graphical Contacts*). Both Graphical and IFR Contacts are fully integrated with SMS so that information about any particular amino acid is highlighted in simultaneous fashion across sequence, structure and contacts window.

The diagram Ramachandran Plot [6], used for checking the quality of the structure, is presented in SMS using all advantages of Java programming language. Menu options on interactive SMS Ramachandran Plot allow for coupling of data displayed in the dihedral angle window with a window showing the 3D structure of a molecule. Number of subsets among amino acids can be highlighted for better correlation of a 3D structure position and a phi-psi spot. Full integration and data coupling makes this SMS component a breed apart from the similar public domain products. A user may also produce an image in the gif format which is more appropriate for printing of publication quality figures. Again, SMS Ramachandran Plot is fully integrated with other SMS windows, allowing a user to concomitantly see structure and sequence information highlighted according to selection done in Ramachandran plot or in the sequence window.

The module *Scorpion *provides a graphical presentation for simple statistical data on a frequency of occurrence for given amino acid and also for amino acid local environment in terms of class of amino acids surrounding given central residue.

The *Protein Dossier *module provides a graphical report of several important structural characteristics of the PDB entry. It offers a plot from PDB cartoon annotated with color coded scales representing for each amino acid a corresponding temperature factor, solvent accessibility of the chain in isolation and in a complex with other present chains in the PDB file, sequence conservation in (HSSP derived) multiple alignment (relative sequence entropy) and histograms representing the atomic contacts (as in the Graphical contacts module), as well as IFR residue identification and IFR contacts. In addition, comparison of the Secondary Structure annotated by PDB, by DSSP [7] and by STRIDE [8] is presented.

With *STINGpaint *it is possible to paint amino acids within multiple alignment of sequences according to two optional color schemes: STING's scheme and William Taylor [9] color scheme. This has effect on how easily the user can grasp regions of sequence identity. In addition, the user is presented with an entropy bar which facilitates even further pinpointing highly variable positions.

The *ConSSeq *presents a sequence for a given PDB file and a consensus sequence (as found in the HSSP). A consensus sequence is obtained from the sequence alignment of the sequence-wise homologous proteins. Above those two sequences, ConSSeq shows a graphic bars colored by scale of colors according to the sequence conservation. The height of graphic bars is reflecting relative entropy. ConSSeq also offers information about residues present in other homologous sequences, with their respective frequency. For fast inspection of data, this program also generates a sequence logo. Complete interactivity with both sequence and chime-structure frame/window of the SMS is now operational, offering much better conditions for the thorough analysis of structure and sequence (alignment) interdependence.

The *Java Cα-Cα [Cβ-Cβ] Distance Plot *is a diagram where the distances between the α [β] carbon of one residue and all α [β] carbon atoms of other residues, within a single chain of the PDB file, are represented by colored squares in a symmetrical plot.

All the above mentioned modules and some others available from SMS, can be accessed either from the STING Millennium's sequence window or entering through the independent entry web page. An extensive list of links is available to increment a volume of information on a protein under the study.

In this new SMS release we introduced ProTherm [10] link, exceptionally important information on protein stability/mutations, provided by the web site of Dr. Akinori Sarai group.

The Sting Millennium and some of the SMS components are now capable of importing local files in PDB format.

### Algorithm and implementation

SMS is organized in two logical layers: SMS server and SMS client. The server side is responsible for updating regularly all relevant public domain databases used by SMS. At the same time, SMS server is also responsible for calculation of a number of macromolecular properties for each PDB structure. The SMS client side provides to a user a friendly graphical interface and communicates to the SMS server, sending user's requests and receiving SMS responses.

SMS interactive interface has been mostly implemented in the Java programming language, taking advantage of its object oriented design and graphical representation capabilities. Most important Java classes in SMS are dedicated to sequence and structure parameter presentation, depiction and interaction. Additional classes are used for efficient data handling utilities. As it is known, the object oriented software design is suitable especially because of its ease in code reusability and also because it provides interfaces for linking new software modules, resulting in systems easily expandable and built with extended capabilities. In addition, the Java programming language is very attractive to users for reasons of portability a key feature in today's versatile computing world. SMS also make extensive use of the C^++ ^programming language, mostly for complex calculation of specific parameters.

SMS runs in the Netscape browser or in Microsoft Internet Explorer (for Microsoft Windows operating system) and in the Netscape/Mozila (for the Linux OS) and requires installation of *Java Plugin 1.3.1 *and CHIME. Some restrictions apply, so a user is invited to consult details of SMS Requirements. Users can run the SMS program by selecting a previously deposited structure in the Protein Data Bank, or using local files with pdb format.

### Input file format for SMS

SMS accepts the PDB format files from RCSB/PDB repository and also accepts local files of the same format, at the client end. A user is able to see structure of the local file in chime/SMS structure window as well as a sequence corresponding to this particular structure. The sequence itself is presented in the separate sequence window. Additionally, some other SMS components will work fine with user's local files: Graphical Contacts, IFR Graphical Contacts, SMS Ramachandran Plot, Scorpion, Formiga, Ca-Ca and Cb-CB contacts and Protein Dossier (although the last one might not have all the usual components that it displays for public PDB files).

### Comparison to other software packages

Increase in availability of molecular structure data during the last decade, urged the development of computer applications for sequence/structure analysis and visualization. Consequently, numerous approaches have been made to the problem of sequence/structure visualization and analysis, resulting in diverse software packages: Protein Explorer, Cn3D, Swiss PdbViewer and ProCheck [11-14]. Each of these products seems to have been developed primarily to accomplish specific tasks. Inevitably, these products have differential strengths in areas that they cover, making difficult the task of comparisons and definitely arbitrary to certain extent.

SMS, as well as comparable software resources, come with intuitive user friendly GUIs, allowing for easy navigation through the vast amount of structural data.

SMS main advantage is the clear presentation of sequence along with the structure in addition to number of visualizing tools for variety of structure describing parameters. In the input layer SMS uses data from public databases: PDB, HSSP, DSSP and SwissProt. Simultaneous display of computed features/parameters/descriptors along with available annotations from above Databanks provides a useful and reach environment, which may complement and in many cases substitute and exceed the already existing tools for sequence/structure/function analysis and visualization.

## Conclusions

Structure analysis is a difficult task due to the large amount of possible parameters/descriptors that can be calculated and associated with the sequence and corresponding structure. The way in which structure data and structure descriptors are stored and displayed, represents a major challenge when interactivity of a user with the data dispersed among many resources is addressed. Several structure viewers already exist, each one of them better suited to different needs and research interests. SMS offers an easy to use computer environment, designed to facilitate concomitant display of as many parameters as possible, coupled in a consistent fashion to each other. Experimental data and calculated information are all embodied in a clear display that offers instantly an intuitive aspect of a given structure and a large amount of biological information at hand. Inspection of the SMS displayed information can lead to valuable conclusions and cover a wide variety of biology issues concerning entire protein families.

SMS has already been applied as a didactic tool for learning details about sequence/structure/function relationship in several universities. Future plans to extend the software platform include the ability to handle ever more descriptors/parameters of protein structure with the simultaneous display and analysis including data extracted from the statistical elaboration of common features among members of certain protein families/folds.

In order to achieve such goal, we count with most generic yet very usable tools: Chime viewer and JAVA programming language. In addition, we count on growing interest of other research groups in participating in this project, contributing with their data and benefiting from the resulting unification of data format and data display. Issues such as the geometrical increase in the volume of the disk space and available CPU time for updating such a large data base should be taken into account.

SMS is available free and can be accessed through the web. A user has to be careful with proper configuration of IT components (Operating System, browser, Chime viewer, Java JER version, firewall warnings) so that SMS can be used to its fullest potential. The detailed online manual/help/tutorial for viewing and analyzing displayed data is available and recommended for frequent consultation.

## Availability and requirements

**Project Name: **STING Millennium Suite

**Lab Home Page: **

**Project Home Page: **

Operating System(s):

Servers: Extensively tested on SGI IRIX 6.5, SUN Solaris7.0 and 8.0 and LINUX Red Hat 7.3, 8.0

Clients: MS Windows XP, NT, 2000 with Netscape 7.0 and IE 6.0 SP1, platform with Java Runtime Environment (JRE) 1.3.1 installed and Linux Rad Hat with Mozila/Netscape 7.0 and CrossOver plugin.

Chime 2.6 SP3/SP4 (depending on OS and browser used) plugin is essential for structure presentation.

**Programming Language: **JAVA, C++, Fortran, JavaScript

**Other requirements: **Installation of JRE 1.3.1.

**License: **Free for Academic use.

## Abbreviations

SMS: Sting Millennium Suite

IFR: Interface Forming Residues

FER: Folding Essential Residue

PDB: Protein Data Bank

RCSB: Research Collaboratory for Structural Bioinformatics

GUI: Graphical User Interface

## Authors' contributions

RH created the Graphical User Interface and part of the data processing programming and general procedures for SMS mirror installation, RT worked on general GUI and statistics for SMS access, AJM worked on ConSSeq integration to SMS, JP worked on HTML design and SMS help pages, IO worked on SMS implementation on Linux OS, PK worked on general data interpretation and GUI suggestions, MY worked on mathematical algorithms for parameter calculation, AM carried out most of the data processing and programming, and GN coordinated the whole project, suggesting the general directions and innovating features of the application. All authors have read and accepted the final manuscript.

## Figures and Tables

**Figure 1:**
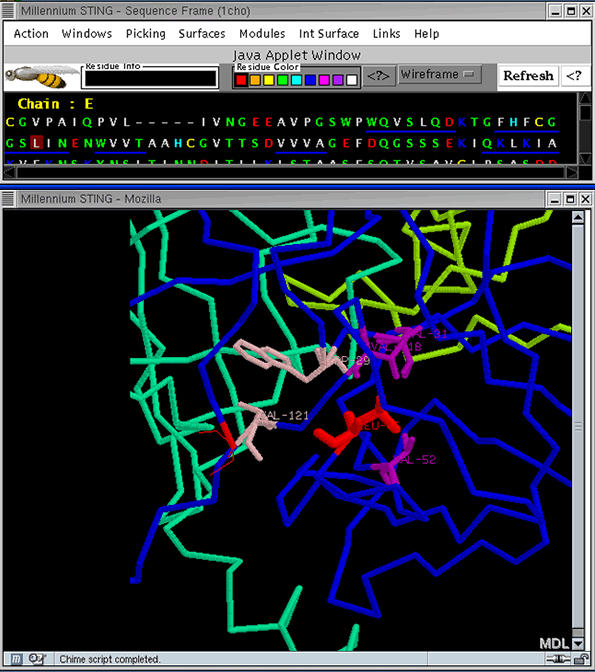
**SMS sequence and structure window. **This SMS example screen displays the pdb file *1cho.pdb*, highlighting the residue Leu46 in chain E. SMS sequence frame, from where all the SMS modules and features can be accessed, is shown at the top of this figure. The sequence is colored according to physical-chemical properties of the amino acids. The blue and red lines underneath the amino acid sequence represent the secondary structure elements (beta strands and helices respectively), according to the pdb file annotation. As the user scrolls the mouse over the sequence, information about the residues appears in the "residue info" box. Pull-down menu on the top of the sequence frame is also shown, demonstrating choice of SMS modules and rendering. SMS structure frame (Chime wiondow) is shown at the bottom of this figure. This figure was centered on the Leu_46 (painted in red) of the E chain. The other residues drawn in stick presentation are the residues that make contacts with the Leucine, colored according to the contacts they make, corresponding to the first two lines of the Protein Dossier in Figure 2.

**Figure 2:**
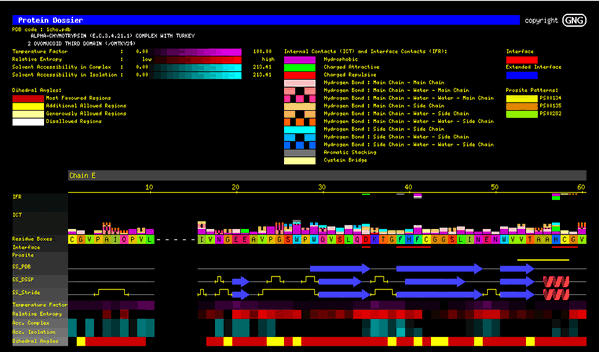
**SMS Protein Dossier. **Protein Dossier module – The meaning of the colors are on the top part of the image. The internal and interface forming contacts are shown above the amino acid sequence cartoon, color coded according to the type of contact. The first row (red line) under the sequence highlights the residues at the interface. The next row (yellow line bellow the sequence stretch: VTAAHGC) indicates the PROSITE pattern PS00134. The following three rows indicate the secondary structure according to the annotations in PDB, DSSP and Stride, respectively. Another five rows are displayed color coded according to: temperature factor, relative entropy, accessibility in complex, accessibility in isolation and dihedral angles.

**Figure 3:**
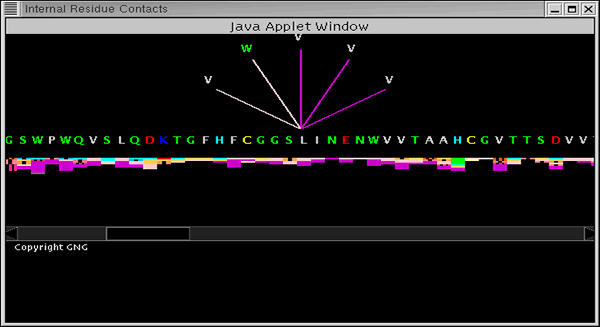
**Atomic contacts among residues within same chain. **Internal residue contacts formed by the Leu_46, showing all amino acids, belonging to the same chain E, that make contacts with it. The fan above the residue is color coded according to the type of contact the residue is involved with.

**Figure 4:**
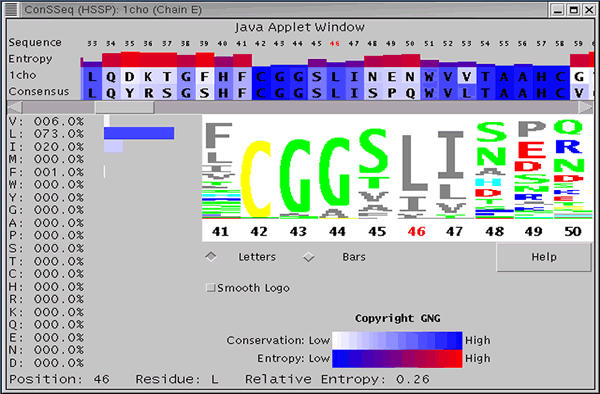
**Sequence Conservation in SMS ConSSeq. **ConSSeq window where the amino acid sequence of 1cho, chain E is aligned with a consensus sequence as found in HSSP and, entropy histogram color and size coded with respect to the calculated degree of conservation. The size of sequence letters in the "logo" follows the same degree of conservation, while color coding is the same as in SMS sequence frame. Leu_46 is highlighted by sequence number 46 colored red.

**Figure 5:**
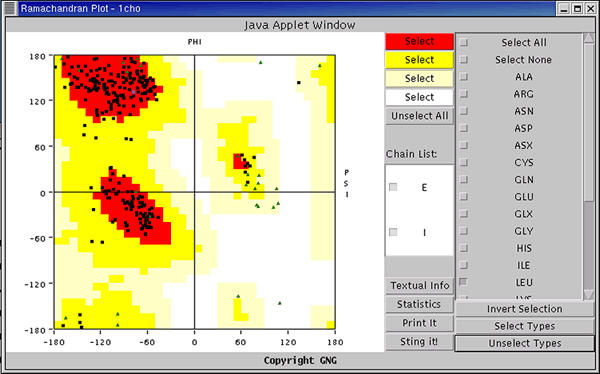
**SMS Ramachandran interactive plot. **Ramachandran plot of *1cho.pdb*, highlighting the position of Leu_46 (blue colored spot), chain E in the diagram, and coloring plotted areas according to the allowed regions for the phi and psi angles. This image shows also the area of the applet which is dedicated to sub setting of the amino acids according to their type and position in the allowed and disallowed areas.

## References

[B1] Berman HM, Westbrook J, Feng Z, Gilliland G, Bhat TN, Weissig H, Shindyalov IN, Bourne PE (2000). The Protein Data Bank. Nucleic Acids Research.

[B2] Sander C, Schneider R (1991). Database of Homology-Derived Protein Structures and the Structural Meaning of Sequence Alignment. Proteins: Struc Func and Genet.

[B3] Schneider R, Sander C (1996). The HSSP database of protein structure sequence alignments. Nucleic Acids Res.

[B4] Bucher P, Bairoch A, Altman R, Brutlag D, Karp P, Lathrop R, Searls D (1994). A generalized profile syntax for biomolecular sequences motifs and its function in automatic sequence interpretation. (In) ISMB-94; Proceedings 2nd International Conference on Intelligent Systems for Molecular Biology.

[B5] Neshich G, Togawa R, Mancini AL, Kuser PR, Yamagishi MEB, Pappas JG, Torres WV, Campos TF, Ferreira LL, Luna FM, Oliveira AG, Miura RT, Inoue MK, Horita LG, de Souza DF, Dominiquini F, Álvaro A, Lima CS, Ogawa FO, Gomes BG, Palandrani JCF, dos Santos GF, de Freitas EM, Mattiuz AR, Costa IC, de Almeida CL, Souza S, Baudet C, Higa RH STING Millennium: a Web based suite of programs for comprehensive and simultaneous analysis of protein structure and sequence. Nucleic Acids Res.

[B6] Ramachandran GN, Ramakrishnan C, Sasisekharan V (1963). Stereochemistry of polypeptide chain configurations. J Mol Biol.

[B7] Kabsch W, Sander C (1983). Dictionary of Protein Secondary Structure: Pattern Recognition of Hydrogen-Bonded and Geometric Features. Biopolymers.

[B8] Frishman D, Argos P (1995). Knowledge-Based Protein Secondary Structure Assignment. Proteins: Struc Func and Genet.

[B9] Taylor WR (1997). Residual colors: a proposal for aminochromography. Protein Eng.

[B10] Abdulla BK, Michael GM, Uedaira H, Kitajima K, Sarai A (2004). ProTherm, version 4.0: Thermodynamic Database for Proteins and Mutants. Nucleic Acids Res.

[B11] Martz E (2002). Protein Explorer: Easy Yet Powerful Macromolecular Visualization. Trends in Bioch Sci.

[B12] Hogue CW (1997). Cn3D: a new generation of three-dimensional molecular structure viewer. Trends in Biochem Sci.

[B13] Guex N, Peitsch MC (1997). SWISS-MODEL and the Swiss-PdbViewer: An environment for comparative protein modeling. Electrophoresis.

[B14] Laskowski RA, MacArthur MW, Moss DS, Thornton JM (1993). PROCHECK: a program to check the stereochemical quality of protein structures. J Appl Cryst.

